# Placental Galectins Are Key Players in Regulating the Maternal Adaptive Immune Response

**DOI:** 10.3389/fimmu.2019.01240

**Published:** 2019-06-19

**Authors:** Andrea Balogh, Eszter Toth, Roberto Romero, Katalin Parej, Diana Csala, Nikolett L. Szenasi, Istvan Hajdu, Kata Juhasz, Arpad F. Kovacs, Hamutal Meiri, Petronella Hupuczi, Adi L. Tarca, Sonia S. Hassan, Offer Erez, Peter Zavodszky, Janos Matko, Zoltan Papp, Simona W. Rossi, Sinuhe Hahn, Eva Pallinger, Nandor Gabor Than

**Affiliations:** ^1^Systems Biology of Reproduction Momentum Research Group, Institute of Enzymology, Research Centre for Natural Sciences, Hungarian Academy of Sciences, Budapest, Hungary; ^2^Department of Immunology, Eotvos Lorand University, Budapest, Hungary; ^3^Perinatology Research Branch, Eunice Kennedy Shriver National Institute of Child Health and Human Development, National Institutes of Health, U.S. Department of Health and Human Services, Bethesda, MD and Detroit, MI, United States; ^4^Department of Obstetrics and Gynecology, University of Michigan, Ann Arbor, MI, United States; ^5^Department of Epidemiology and Biostatistics, Michigan State University, East Lansing, MI, United States; ^6^Center for Molecular Medicine and Genetics, Wayne State University, Detroit, MI, United States; ^7^Structural Biophysics Research Group, Institute of Enzymology, Research Centre for Natural Sciences, Hungarian Academy of Sciences, Budapest, Hungary; ^8^Department of Genetics, Cell and Immunobiology, Semmelweis University, Budapest, Hungary; ^9^TeleMarpe Ltd, Tel Aviv, Israel; ^10^Maternity Private Clinic of Obstetrics and Gynecology, Budapest, Hungary; ^11^Department of Obstetrics and Gynecology, Wayne State University School of Medicine, Detroit, MI, United States; ^12^Department of Computer Science, Wayne State University College of Engineering, Detroit, MI, United States; ^13^Department of Physiology, Wayne State University School of Medicine, Detroit, MI, United States; ^14^Division of Obstetrics and Gynecology, Maternity Department “D”, Faculty of Health Sciences, Soroka University Medical Center, School of Medicine, Ben Gurion University of the Negev, Beer-Sheva, Israel; ^15^Department of Obstetrics and Gynecology, Semmelweis University, Budapest, Hungary; ^16^Department of Biomedicine, University and University Hospital Basel, Basel, Switzerland; ^17^First Department of Pathology and Experimental Cancer Research, Semmelweis University, Budapest, Hungary

**Keywords:** angiogenesis, glycomics, immune privilege, PP13, trophoblast differentiation, trophoblast invasion

## Abstract

Galectins are potent immunomodulators that regulate maternal immune responses in pregnancy and prevent the rejection of the semi-allogeneic fetus that also occurs in miscarriages. We previously identified a gene cluster on Chromosome 19 that expresses a subfamily of galectins, including galectin-13 (Gal-13) and galectin-14 (Gal-14), which emerged in anthropoid primates. These galectins are expressed only by the placenta and induce the apoptosis of activated T lymphocytes, possibly contributing to a shifted maternal immune balance in pregnancy. The placental expression of Gal-13 and Gal-14 is decreased in preeclampsia, a life-threatening obstetrical syndrome partly attributed to maternal anti-fetal rejection. This study is aimed at revealing the effects of Gal-13 and Gal-14 on T cell functions and comparing the expression of these galectins in placentas from healthy pregnancies and miscarriages. First-trimester placentas were collected from miscarriages and elective termination of pregnancies, tissue microarrays were constructed, and then the expression of Gal-13 and Gal-14 was analyzed by immunohistochemistry and immunoscoring. Recombinant Gal-13 and Gal-14 were expressed and purified, and their effects were investigated on primary peripheral blood T cells. The binding of Gal-13 and Gal-14 to T cells and the effects of these galectins on apoptosis, activation marker (CD25, CD71, CD95, HLA-DR) expression and cytokine (IL-1β, IL-6, IL-8, IL-10, IFNγ) production of T cells were examined by flow cytometry. Gal-13 and Gal-14 are primarily expressed by the syncytiotrophoblast at the maternal-fetal interface in the first trimester, and their placental expression is decreased in miscarriages compared to first-trimester controls. Recombinant Gal-13 and Gal-14 bind to T cells in a population- and activation-dependent manner. Gal-13 and Gal-14 induce apoptosis of Th and Tc cell populations, regardless of their activation status. Out of the investigated activation markers, Gal-14 decreases the cell surface expression of CD71, Gal-13 increases the expression of CD25, and both galectins increase the expression of CD95 on T cells. Non-activated T cells produce larger amounts of IL-8 in the presence of Gal-13 or Gal-14. In conclusion, these results show that Gal-13 and Gal-14 already provide an immunoprivileged environment at the maternal-fetal interface during early pregnancy, and their reduced expression is related to miscarriages.

## Introduction

The mechanisms sustaining maternal immune tolerance to the semi-allogeneic fetus while shielding against microbial infections during pregnancy as well as the changes and interplay of maternal, fetal, and placental immune responses during pregnancy are of major interest in reproductive research (1–41). These immune tolerance mechanisms are complex and dynamic given that implantation involves decidual inflammation; the second trimester of pregnancy is characterized by a predominantly anti-inflammatory milieu in the womb, while at the end of the third trimester, the initiation of parturition requires a transition toward physiologic pro-inflammatory responses (42–44). Recent evolutionary evidence has shown that the pro-inflammatory implantation reaction in humans, as in all eutherian mammals, is derived from an inflammatory attachment reaction in the uterus of the ancestral therian mammals that directly leads to parturition, and that a key innovation in eutherian mammals was the shift from this inflammatory attachment reaction to the non-inflammatory mid-pregnancy period, which allowed an extended period of intimate placentation (45, 46). Although the molecular changes of this evolutionary shift in uterine immune responses are not yet explored in detail, these may include the placental expression of molecules that down-regulate maternal immune responses (47–67). This is substantiated by the fact that the dysregulated expression of immunoregulatory molecules at the maternal-fetal interface and the consequent disturbances in maternal-fetal immune regulation and pro-inflammatory processes are associated with the development of the great obstetrical syndromes, including miscarriage (68–71), preterm labor (72–80), or preeclampsia (81–87).

Regulation of the immune system is mediated by a complex network of cellular and molecular interactions, including glycan recognition by endogenous lectins (61, 88–90). Galectins, a subfamily of lectins specifically bind β-galactoside-containing glycoconjugates, also on immune cell surfaces where they modify immune responses by cross-linking receptors (61, 89, 91–93). Galectins have pleiotropic functions given their binding to a diverse set of cell surface ligands on immune and other cells including trophoblasts (94, 95). In mammals, 19 galectins have been identified, of which 13 are expressed in human tissues (56, 61, 92, 93). Studies of past decades began the exploration of the diverse functions of human galectins, primarily galectins-1, -3, and -9, in innate and adaptive immune responses including the regulation of leukocyte homing, adhesion, apoptosis, pathogen sensing, and immune signaling, also observed in reproductive processes (52, 96–102). Of major interest, several human galectins have an abundant expression at the maternal-fetal interface (31, 52, 53, 56, 58, 97, 103–109), and galectins-13, -14, and -16 are solely expressed by the human placenta (53, 56, 58, 61). These three galectins are expressed from a gene cluster on Chromosome 19 that had emerged in anthropoid primates (53, 56, 61, 110).

We recently started to explore the biological functions of Chromosome 19 galectins in pregnancy (53). Galectin-13 and galectin-14 (Gal-13 and Gal-14), originally described as placental protein 13 (PP13) (111) and placental protein 13-like (PPL13) (112), respectively, are strongly expressed in the syncytiotrophoblast at the lining of the maternal-fetal interface (53, 106, 110, 113, 114). The expression of these galectins is dependent on trophoblast differentiation, and this developmentally regulated process in the trophoblast emerged during primate evolution (110). Of importance, Gal-13 is secreted from the syncytiotrophoblast into the maternal circulation, and low Gal-13 concentration in the maternal circulation in the first trimester was found in women who subsequently developed preterm preeclampsia (115–122), a severe obstetrical syndrome with a strong systemic immune dysregulation (51, 82, 86, 123–128) that already exists in the first trimester (129). Our studies have also shown that the placental expression of Gal-13 and Gal-14 is down-regulated in preterm preeclampsia (81, 110, 113, 129), where the placental pathology and the pro-inflammatory changes are similar to that of miscarriage (130–135).

Since we and our collaborators have shown that Gal-13 and Gal-14 induce the apoptosis of pre-activated T lymphocytes (53) and that Gal-13 increased IL-1α and IL-6 secretion from peripheral blood mononuclear cells (PBMCs) in pregnant women (114), an unanswered question remained: are Gal-13 and Gal-14 critical regulators of immune processes at the early maternal-fetal interface that can be considerably dysregulated in miscarriage? Therefore, we investigated the placental expression of Gal-13 and Gal-14 in miscarriage and also the effects of Gal-13 and Gal-14 on human T lymphocyte functions, which may play a critical role in immune tolerance and rejection. Indeed, we show herein that these placenta-specific galectins moderate adaptive immune responses and are down-regulated in miscarriages, suggesting that their reduced expression is related to the immunopathology of miscarriage.

## Materials and Methods

### Study Groups, Clinical Definitions, and Sample Collection

Placental tissue samples, collected from Caucasian women, were processed immediately after sample collection as previously described (81, 136), fixed in 10% neutral-buffered formalin, and were then embedded in paraffin (FFPE). First- (*n* = 40) and third- (*n* = 2) trimester placentas were collected prospectively at the Maternity Private Department, Semmelweis University (Budapest, Hungary). Pregnancies were dated according to ultrasound scans collected between 5 and 13 weeks of gestation. Patients with a twin gestation were excluded. Women were enrolled in two groups: those who underwent elective termination of pregnancy (control, *n* = 30) and those who miscarried their pregnancy (cases, *n* = 10) (Table 1). Miscarriage was defined according to the American College of Obstetricians and Gynecologists Practice Bulletin, as a non-viable, intrauterine pregnancy with a gestational sac containing an embryo or fetus without fetal heart activity within the first 12 6/7 weeks of gestation (137).

**Table 1:** Demographic and clinical data of the first-trimester placental study groups.

**Groups**	**Control**	**Miscarriage**
Number of cases^a^	30	10
Maternal age (years)^b^	30 (19–41)	36 (27–42)^*^
Gestational age at surgery (weeks)^b^	8.7 (5.0–11.9)	9.4 (7.0–13.0)
Gravidity^b^, ^d^	2 (1–6)	2 (1–7)
Parity^b^, ^d^	0 (0–4)	0 (0–1)
Habitual abortion^c^, ^d^	3	30^**^

All women were Caucasian

a *Values are presented as numbers*.

b *Values are presented as medians (interquartile (IQR) range)*.

c *Values are presented as a percentages*.

d *Data were available for 29 cases in the control group*.

* *p < 0.05 compared to gestational age-matched controls*.

** *p < 0.01 compared to gestational age-matched controls*.

Clinical samples and data collection were approved by the Health Science Board of Hungary (ETT-TUKEB 4834-0/2011-1018EKU). Written informed consent was obtained from women prior to sample collection and the experiments conformed to the principles set out in the World Medical Association Declaration of Helsinki. Specimens and data were stored anonymously.

### Histopathologic Evaluation of the Placentas

Five-micrometers-thick sections were cut from FFPE tissue blocks and stained with hematoxylin and eosin for histopathological evaluation at the 1st Department of Pathology and Experimental Cancer Research, Semmelweis University. The sections were examined using light microscopy by a perinatal pathologist blinded to the clinical information. Histopathologic changes were defined according to published criteria (136, 138, 139).

### Tissue Microarray Construction, and Galectin-13 and Galectin-14 Immunostainings

As previously described (140–143), representative areas were selected for the construction of tissue microarrays (TMAs), which contained 2 mm cores in diameter. To investigate protein expressions, two TMAs were created, using an automated tissue arrayer (TMA Master II, 3DHISTECH Ltd.), to contain one block of each first-trimester (*n* = 40) placenta as well as a positive control (third-trimester healthy placenta) and a negative control (liver) in triplicate.

Five-micrometers-thick sections were cut from TMAs and placed on silanized slides. After deparaffinization and rehydration, antigen retrieval was performed using citrate buffer (10 mM Sodium citrate, 0.05% Tween 20, pH = 6) for 5 min at 100°C in a pressure cooker. Endogen peroxidase blocking was performed using 10% H_2_O_2_ for 20 min. Immunostaining was carried out using the Novolink Polymer Detection System (Novocastra Laboratories), according to the manufacturer's protocol, as detailed in Supplementary Table 1. Slides were blocked for 10 min with Protein Block. To evaluate Gal-13 expression, slides were incubated with anti-galectin-13 mouse monoclonal antibody (clone 215-28-3) in 1% BSA-TBS for 60 min at 37°C. To evaluate Gal-14 expression, slides were incubated with anti-galectin-14 recombinant human antibody in 1% BSA-TBS for 60 min at room temperature. In the case of Gal-14 staining, after three washes with Tris buffer saline with 0.05% Tween 20 (TBST), slides were incubated with anti-His_6_ mouse monoclonal antibody for 30 min at room temperature. In both circumstances, subsequent steps were the same. Briefly, after three washes with TBST and Post Primary treatment (30 min, at room temperature), Novolink Polymer was used as the secondary antibody for 30 min at room temperature. This was followed by three washes with TBST, and then the sections were developed using 3,3′-diaminobenzidine (DAB, Novolink) in 1:20 dilution. Finally, sections were counterstained with hematoxylin, and these were mounted with DPX Mountant (Sigma-Aldrich) after dehydration.

### Evaluation of Immunostainings

Gal-13 or Gal-14 immunostained placental TMAs were digitally scanned by a high-resolution bright field slide scanner (Pannoramic Scan, 3DHISTECH Ltd.), and cytoplasmic staining in the syncytiotrophoblast was evaluated on virtual slides using Pannoramic Viewer 1.15.4 (3DHISTECH Ltd.) by two examiners blinded to the clinical information. All villi were scored semi-quantitatively. The intensity of immunostaining was graded from 0 to 3. The average intensity was determined for each core as the representative data for that core. By averaging immunoscores of the cores, the overall intensity score was assigned to each placenta and then to each patient group.

### Expression and Purification of Recombinant Galectin-13 and Galectin-14

Recombinant Gal-13/Gal-14 was expressed as previously described (53) with modifications. Expression plasmids used earlier (53) were modified by the N-terminal insertion of maltose-binding protein (MBP) tag on these galectins. These modified plasmids, containing either full-length Gal-13 or Gal-14 as well as N-terminal maltose-binding protein (MBP)- and C-terminal His_6_-tags, were transformed into ClearColi BL21 (DE3) (Lucigen). For protein expression, cells were grown in LB-Miller broth to OD_600_ = 0.6 at 37°C, induced with 0.4 mM isopropyl β-D-1-thiogalactopyranoside (IPTG), and further grown for 4 h at 30°C. The following purification steps were applied: affinity purification on MBPTrap HP column (GE Healthcare Life Sciences); size exclusion chromatography (Superdex 200 Increase SEC column, GE Healthcare Life Sciences) for elimination of aggregates (only for Gal-14); MBP cleavage by Tobacco Etch virus (TEV) protease [expressed and purified according to Kapust et al. (144)]; affinity chromatography on HisTrap HP columns (GE Healthcare Life Sciences); desalting and buffer exchange on Bio-Gel P-6 Desalting Cartridge (Bio-Scale Mini, Bio-Rad). All steps were carried out in the presence of 1 mM dithiothreitol (DTT). Finally, Gal-13 and Gal-14 in PBS, supplemented with 1 mM DTT, were aliquoted and stored at −80°C.

### Checking the Purity and Carbohydrate Binding Properties of Recombinant Gal-13 and Gal-14

The purity of the recombinant galectins was verified by heating the samples in Laemmli buffer for 10 min at 70°C, followed by 15% SDS polyacrylamide gel electrophoresis (SDS-PAGE) (Bio-Rad). After gel electrophoresis, recombinant galectins were either subjected to Coomassie blue staining (Supplementary Figure 1A) or transferred to nitrocellulose membranes. Membranes were blocked with 5% non-fat dry milk in TBST for 1 h, and then these were incubated overnight at 4°C with primary antibodies to Gal-13 (clone 27-3-2) or Gal-14 in TBST with 5% BSA. After repeated washing with TBST, blots were incubated for 1 h with HRP-goat anti-mouse IgG antibody (ThermoFisher Scientific) for Gal-13 or with HRP-goat anti-human IgG F(ab')2 antibody (Bio-Rad) for Gal-14 (Supplementary Table 2). After repeated washing with TBST, protein bands were visualized by enhanced chemiluminescence (ECL; Amersham International) (Supplementary Figure 1B).

To determine their functional activity, the binding of purified galectins to asialofetuin (ASF), a naturally occurring multivalent glycoprotein serving as a ligand for several galectins, was assayed by ELISA (145, 146). Briefly, ASF (50 μL of 10 μg/mL bovine ASF in sodium carbonate buffer pH = 9.6) was immobilized in microtiter plates overnight. After blocking residual binding sites with BSA (5% in PBS-Tween, PBST) for 2 h, different amounts of Gal-13 or Gal-14 were incubated for 1 h in PBST with 0.5% BSA. Washing with PBS was done three times between the incubation steps. Bound galectins were detected by incubation with anti-His_6_-HRP antibody in PBST with 0.5% BSA (Biolegend, 1:1,000) and by the subsequent conversion of 3,3′5,5′-tetramethylbenzidine (TMB; Sigma-Aldrich) with a readout at 450 nm (reference filter: 620 nm). The reaction was stopped by 4N H_2_SO_4_ (Supplementary Figure 1C). Additionally, 50 μg/mL recombinant galectins were pre-incubated with gentle rotation on lactose-agarose beads (Sigma-Aldrich) for 1 h at room temperature prior to performing ELISA to also check for lactose inhibition. The inhibition was moderate for Gal-13 and weak for Gal-14, as we found differential binding of Gal-13 and Gal-14 to lactose and other carbohydrates in a previous study (53).

### Isolation of Primary Immune Cells

Blood samples were obtained from a donor pool of non-pregnant, healthy, human females (*n* = 18 in total, *n* = 4–8 per assay, median age: 29.5) who were in the pre-ovulatory phase. PBMCs were isolated by Ficoll-Hypaque (Sigma-Aldrich) density-gradient centrifugation and washed in RPMI 1640 medium (ThermoFisher Scientific) before experimentations. T cells were isolated from PBMCs with the Dynabeads untouched human T cell kit (ThermoFisher Scientific) according to the manufacturer's protocol. PBMCs or T lymphocytes were kept in RPMI 1640 medium supplemented with 10% FBS and gentamycin or were activated for 48/72 h with the Dynabeads human T-Activator CD3/CD28 (ThermoFisher Scientific), according to the manufacturer's instructions, before treatment with Gal-13 or Gal-14 for 24 h.

### Binding of Gal-13 and Gal-14 to Peripheral Blood T Cells

Fresh PBMCs from three donors, or PBMCs activated or not with human T-Activator for 72 h, were used for the Gal-13/Gal-14 binding study. To measure the binding of recombinant Gal-13 or Gal-14 to the surface of T cells, 2 ×10^5^ PBMCs were initially washed in PBS containing 1% BSA. Recombinant Gal-13 or Gal-14 (4 μM), which we conjugated with CF488 fluorophore using the Mix-n-Stain CF488 kit (Sigma-Aldrich) according to the technical bulletin, was added to the cells, and samples were incubated for 45 min on ice. After washing, Fc receptors were blocked with human FcR blocking reagent (Miltenyi Biotec) for 5 min on ice. Anti-CD3-APC, anti-CD4-PerCP, and anti-CD8-APC/Fire750 antibodies (Biolegend) were applied to discriminate between T lymphocyte populations. All antibodies and reagents are listed in Supplementary Table 3. Flow cytofluorimetric measurements were carried out on a CytoFLEX device (Beckman Coulter) by collecting data from 50,000 cells. Data were analyzed using FlowJo v10 software (FlowJo, LLC).

### Apoptosis Assay

PBMCs (5 ×10^5^), previously activated or not with human T-Activator for 48 h, were incubated for 24 h on tissue culture plates with 0.25 or 4 μM recombinant Gal-13 or Gal-14 in RPMI 1640 medium supplemented with 10% FBS. The 10^5^ cells were stained with anti-CD3-APC and anti-CD8-FITC antibodies, as described above. Cells were then incubated in 100 μL of annexin-binding buffer containing phycoerythrin-conjugated Annexin V (Annexin V-PE) and 7-amino-actinomycin D (7-AAD) (Annexin-V Apoptosis Detection Kit, ThermoFisher Scientific; Supplementary Table 3) for 15 min at room temperature in the dark. After incubation, 400 μL annexin binding buffer was added, and samples were measured immediately on a FACSCalibur cytofluorimeter using Cell Quest software (BD Biosciences). The Annexin V-PE^−^/7-AAD^−^ population was regarded as normal, while the Annexin V-PE^+^/7-AAD^−^ and Annexin V-PE^+^/7-AAD^+^ populations were taken as measurements of early and late apoptotic cells, respectively. Data were analyzed using FlowJo v10 software.

### Flow Cytometry Measurement of Activation Markers

The PBMCs (5 ×10^5^), previously activated or not with human T-Activator for 72 h, were incubated for 24 h on tissue culture plates with 4 μM recombinant Gal-13 or Gal-14 in RPMI 1640 medium supplemented with 10% FBS. To examine cell surface markers, 2 ×10^5^ PBMCs were initially washed in PBS containing 1% FBS. Fc receptors were blocked with human FcR blocking reagent for 5 min on ice; then, specific antibodies to mid-late and late activation markers CD25 (Interleukin-2 Receptor alpha, IL-2Rα), CD71 (Transferrin Receptor, TfR), CD95 (Fas Cell Surface Death Receptor, Fas), and HLA-DR (Human Leukocyte Antigen, DR isotype; member of MHC-II) were added to the cells. Anti-CD3-APC and anti-CD8-FITC antibodies were added simultaneously and samples were incubated for 20 min on ice. All antibodies are listed in Supplementary Table 3. After washing, cells were measured in a CytoFLEX flow cytofluorimeter. A total of 20,000 cells were collected and data were analyzed using FlowJo v10 software.

### Measurement of Cytokine Production by Bead Array

The T lymphocytes were isolated as described above. The 5 ×10^5^ cells, previously activated or not with human T-Activator for 72 h, were incubated for 24 h on tissue culture plates with 4 μM recombinant Gal-13 or Gal-14 in RPMI 1640 medium supplemented with 10% FBS. Supernatants were collected in all cases, centrifuged at 400 g for 10 min, aliquoted and stored at −80°C until use. LEGENDplex bead-based immunoassays (Biolegend) were applied to measure the concentration of IL-8, IL-10, IFNγ, IL-1β, and IL-6 cytokines in cell culture supernatants of T cells, according to the manufacturer's instruction. Beads were measured in a FACSCalibur flow cytofluorimeter and data were analyzed using FlowJo v10 software.

### Statistical Analysis

Statistical analysis was performed using GraphPad Prism 5.0 (GraphPad Software). An unpaired *t*-test with or without Welch's correction was used to analyze demographic data. An unpaired *t*-test was also used to analyze Gal-13 and Gal-14 immunostainings when comparing first-trimester control and miscarriage groups. The Fisher's exact test was performed to test the distribution of Gal-13 or Gal-14 immunoscores between the control and miscarriage groups. Repeated ANOVA tests with Tukey's *post-hoc* test were used for the analysis of galectin binding and CD4:CD8 ratio upon different treatments. One sample *t*-test was used to compare apoptosis of the Gal-13- and Gal-14-treated groups to the PBS-DTT-treated group, and to analyze the binding of Gal-13 and Gal-14 to ASF with or without lactose pre-treatment. Repeated ANOVA tests with Dunnett's *post-hoc* test were used to compare the non-treated group with Gal-13/Gal-14-treated groups in activation marker expression and cytokine production studies. Results were considered statistically significant at ^*^p < 0.05, ^**^p < 0.01, and ^***^p < 0.001.

## Results

### Gal-13 and Gal-14 Are Expressed in First-Trimester Placentas and Their Expression Is Decreased in Miscarriage

**Figure 1:**
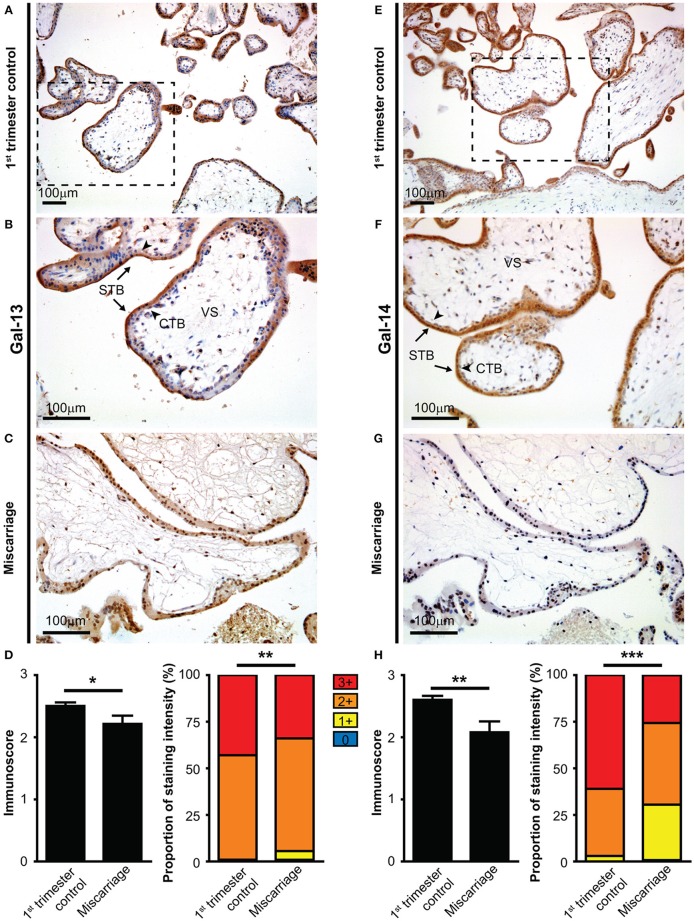
The syncytiotrophoblast expresses galectin-13 and galectin-14 in the first-trimester placenta, which is decreased in miscarriage. Five-micrometers-thick first-trimester placental sections from normal pregnancy **(A,B,E,F)** or from miscarriage **(C,G)** were stained for Gal-13 **(A–C)** or Gal-14 **(E–G)** by specific monoclonal antibodies. Chorionic villi exhibited intense syncytiotrophoblast cytoplasmic staining (arrows, STB), while the villus stroma (VS) and cytotrophoblasts were negative (arrowheads, CTB). Representative images, hematoxylin counterstain, 100x **(A,E)** and 200x **(B,C,F,G)** magnifications. Gal-13 **(D)** and Gal-14 **(H)** immunoscores (mean ± SEM) and proportion of staining intensities in control placentas (*n* = 30) and placentas with miscarriage (*n* = 10) are displayed on left and right graphs, respectively (Gal-13: n_totalvillus_ = 775 and n_totalvillus_ = 106, respectively; Gal-14: n_totalvillus_ = 797 and n_totalvillus_ = 121, respectively). Unpaired *t*-test was used for the comparison of the mean immunoscores of the two groups. Fisher's exact test was performed to test the frequency difference of Gal-13 or Gal-14 immunostaining between control and miscarriage groups (^*^p < 0.05, ^**^p < 0.01, ^***^p < 0.001).

Immunostainings of TMAs revealed that Gal-13 (Figures 1A,B) and Gal-14 (Figures 1E,F) are predominantly expressed in the cytoplasm of the syncytiotrophoblast of chorionic villi in the first trimester, and there were no stainings in the cytotrophoblasts and villous stroma, similar to later stages of pregnancy (53, 81, 106, 110, 113). Moreover, chorionic villi exhibited more intense syncytiotrophoblast cytoplasmic staining in the first trimester than in the third trimester (Supplementary Figure 2). The specificity of the galectin antibodies was confirmed by previous studies and by the lack of Gal-13 and Gal-14 immunostaining of human livers on our TMAs.

Next, we examined whether the expression of Gal-13 and Gal-14 is dysregulated in first-trimester placentas obtained from women who miscarried, as a potential sign of fetal rejection. There was no significant change in Gal-13 or Gal-14 immunoscores with gestational age in control placentas (*R*^2^ = 0.0078 for Gal-14; *R*^2^ = 10^−5^ for Gal-13). However, the average immunoscore of syncytiotrophoblast decreased by 11.5% for Gal-13 (*p* = 0.027, Figures 1A–D) and by 20% (*p* = 0.001) for Gal-14 (Figures 1E–H) in miscarriages compared to gestational age-matched controls. Also, there was a significant difference in the distribution of Gal-13 and Gal-14 immunoscores (*p* = 0.002 and *p* < 0.001, respectively) between the disease and control groups (Figures 1D,H).

### Gal-13 and Gal-14 Bind to Peripheral Blood T Cells

**Figure 2:**
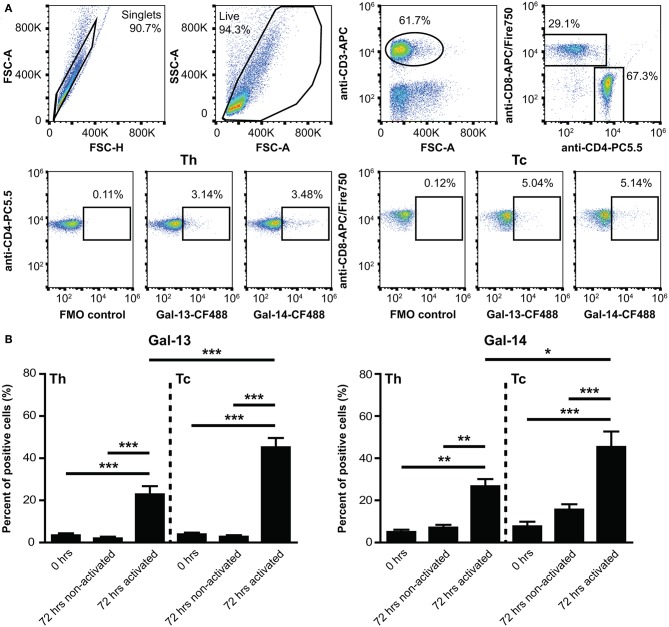
Binding of galectin-13 and galectin-14 to T lymphocytes is population- and activation-dependent**. (A)** Detection of Gal-13 or Gal-14 binding to human T lymphocytes was achieved by flow cytometry with the following cells: freshly isolated PBMCs (0 h) or PBMCs, kept in culture for 72 h in the presence (72 h activated) or absence (72 h non-activated) of anti-CD3/CD28 microbeads. Cells were incubated with 4 μM Gal-13-CF488 or Gal-14-CF488 for 45 min on ice. PBMCs were also stained for CD3 (anti-CD3-APC), CD4 (anti-CD4-PC5.5), and CD8 (anti-CD8-APC/Fire750), in order to distinguish between helper (Th) and cytotoxic (Tc) T lymphocytes. The gating strategy is shown in **(A)**. **(B)** Graphs show the percentage of cells, to which Gal-13 (left) or Gal-14 (right) were bound, as mean ± SEM. Repeated ANOVA with Tukey's *post-hoc* test was used for the comparison of groups (^*^p < 0.05, ^**^p < 0.01, ^***^p < 0.001). Four non-pregnant female donors were included in each group. FMO, Fluorescence minus one; PBMCs, peripheral blood mononuclear cells.

As Gal-13 and Gal-14 are released from the placenta into the maternal circulation, where they may regulate maternal T lymphocytes (53), we further characterized their effects on T cell populations. First, we examined the binding of fluorescent Gal-13 or Gal-14 to primary T cells. We found that Gal-13 and Gal-14 bound to freshly isolated T lymphocytes, either to helper (Th: 3.4 ± 0.9% and 5.1 ± 1%, respectively) or cytotoxic T (Tc: 3.9 ± 0.8% and 7.7 ± 2.2%, respectively) cells (Figures 2A,B, Supplementary Figure 3). Upon activation, binding of Gal-13 to Th and Tc cells was increased by 21% (*p* < 0.001) and 42% (*p* < 0.001), respectively (Figure 2B, Supplementary Figure 3B). Binding of Gal-14 was increased by 20% (*p* < 0.01) and by 30% (*p* < 0.001) to activated Th and Tc cells, respectively, compared to non-activated ones (Figure 2B, Supplementary Figure 3B). Of note, both galectins tended to bind more to Tc over Th lymphocytes, a difference that reached statistical significance in the case of activated cells (*p* < 0.001 for Gal-13 and *p* < 0.05 for Gal-14).

### Gal-13 and Gal-14 Increase Apoptosis of Non-activated and Activated T Cells

**Figure 3:**
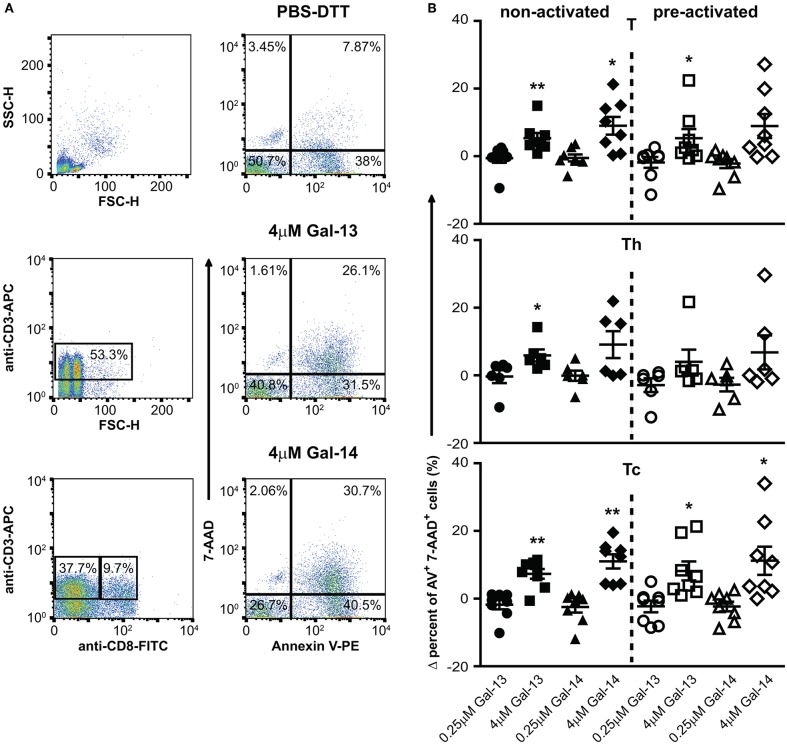
Galectin-13 and galectin-14 increase apoptosis of T lymphocytes**. (A)** PBMCs were kept in culture for 48 h in the presence (activated) or absence (non-activated) of anti-CD3/CD28 microbeads, then treated with Gal-13 or Gal-14 for 24 h. To detect apoptosis of T lymphocytes by flow cytometry, cells were stained with Annexin V-PE and 7-AAD and were also stained for CD3 (anti-CD3-APC), and CD8 (anti-CD8-FITC), in order to distinguish between helper (Th) and cytotoxic (Tc) T cells, respectively. **(A)** The gating strategy (left: CD3^+^ T gate, CD3^+^CD8^+^ Tc gate, CD3^+^CD8^−^ Th gate) and representative Annexin V and 7-AAD dot plots of vehicle- (PBS-DTT), Gal-13- or Gal-14-treated T cells (CD3^+^) are displayed. **(B)** Graphs show the Δ percentage of double positive (Annexin V-PE^+^ 7-AAD^+^) cells as mean ± SEM. One sample t-test was used for the comparison of galectin-treated groups with the PBS-DTT-treated group of non-activated or pre-activated cells (^*^p < 0.05, ^**^p < 0.01). Six-eight non-pregnant female donors were included in each group. PBMCs, Peripheral blood mononuclear cells.

Since certain galectins can induce the apoptosis of T cells depending on T cell subsets and their activation status (147–149), we investigated the effects of Gal-13 and Gal-14 on various T cell populations, either in an activated or a non-activated state. Flow cytometry results, overall, show that 4 μM, but not 0.25 μM, of Gal-13 or Gal-14 increased apoptosis of T lymphocytes either pre-activated or not (Figures 3A,B, Supplementary Figure 4A). Gal-13 increased apoptosis of both non-activated and pre-activated T cells by 5.3% (*p* = 0.010) and 9%, (*p* = 0.011), respectively. Gal-14 increased apoptosis of pre-activated T cells by 8.9% (*p* = 0.040) compared to PBS-DTT treated cells. We further analyzed Th and Tc lymphocytes separately, based on CD3 and CD8 expression. Tc cell apoptosis was increased for both galectins regardless of the activation state (Gal-13, non-activated: 3.9%, *p* = 0.002; Gal-13, pre-activated: 8.2%, *p* = 0.022; Gal-14, non-activated: 11%, *p* = 0.001; Gal-14, pre-activated: 11.2%, *p* = 0.032), while Gal-13 increased apoptosis rate (8.3%, *p* = 0.031) of non-activated Th cells (Figures 3A,B). The proportion of early apoptotic (Annexin V^+^ 7-AAD^−^) T lymphocytes, Th cells, and Tc cells as well did not change upon Gal-13 or Gal-14 treatment (Supplementary Figure 4B).

### Gal-13 and Gal-14 Treatment Alters Cell Surface Expression of T Cell Activation Markers

**Figure 4:**
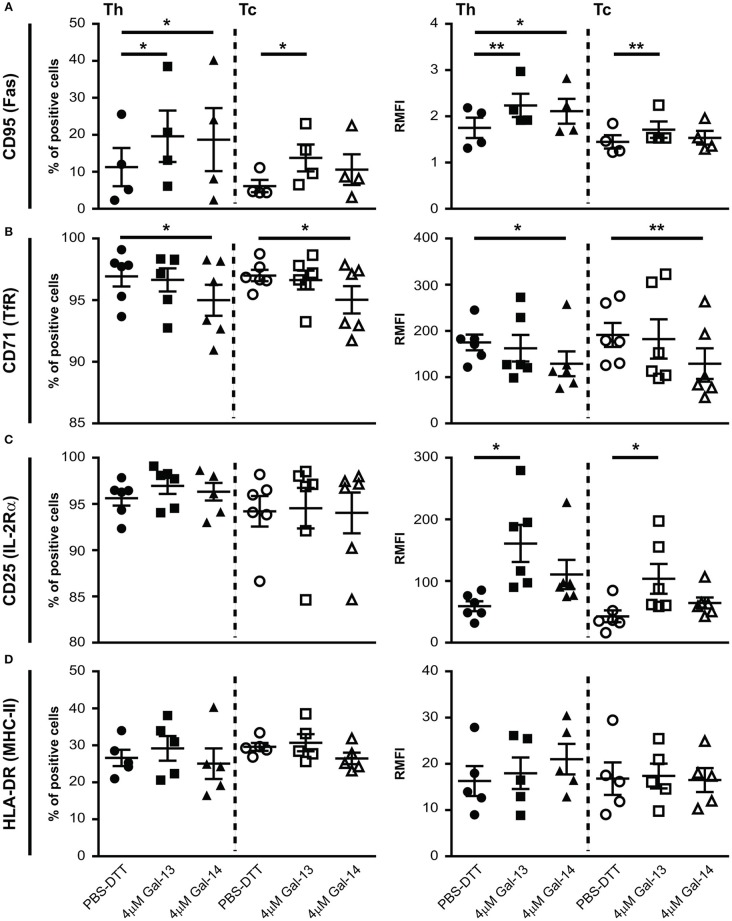
Galectin-13 and galectin-14 affect cell surface expression of activation markers on T lymphocytes. PBMCs were kept in culture for 72 h in the presence of anti-CD3/CD28 microbeads, then treated with Gal-13 or Gal-14 for 24 h. To detect cell surface expression of CD95 **(A)**, CD71 **(B)**, CD25 **(C)**, HLA-DR **(D)** activation markers on T lymphocytes by flow cytometry, cells were stained with anti-CD25-PE and anti-CD71-PerCP/5.5, or anti-CD95-PE and anti-HLA-DR-PerCP/5.5. Cells were also stained for CD3 (anti-CD3-APC), and CD8 (anti-CD8-FITC) in order to distinguish between helper (Th) and cytotoxic (Tc) T cells. Left graphs show the percentage of positive cells and right graphs show relative median fluorescence intensity (RMFI) values (mean ± SEM). RMFI was calculated by dividing specific median fluorescence intensity with the median fluorescence intensity of the isotype control. Repeated ANOVA with Dunnett's *post-hoc* test was used for comparison of the non-treated group with Gal-13/Gal-14-treated groups (^*^p < 0.05, ^**^p < 0.01). Four-six non-pregnant female donors were included in each group. HLA-DR, Human leukocyte antigen DR isotype; PBMCs, peripheral blood mononuclear cells.

Next, we investigated the impact of Gal-13 and Gal-14 on the expression of well-known activation markers—CD25 (IL-2Rα), CD71 (TfR), CD95 (Fas), and HLA-DR (MHC-II)—of T lymphocytes. Interestingly, Gal-13 treatment increased both the percentage of CD95 positive cells and the cell surface expression of CD95 on Th (% control: 11.3 ± 5.2%, Gal-13: 19.6 ± 7%, *p* < 0.05; RMFI control: 1.7 ± 0.2, Gal-13: 2.2 ± 0.2, *p* < 0.01) and Tc (% control: 6.1 ± 1.7%, Gal-13: 13.7 ± 3.7%, *p* < 0.05; RMFI control: 1.4 ± 0.1, Gal-13: 1.7 ± 0.2, *p* < 0.01) lymphocytes. Gal-14 treatment increased the percentage of CD95 positive cells (18.7 ± 8.5%, *p* < 0.05) and the cell surface expression of CD95 on Th (2.1 ± 0.3, *p* < 0.05) but not on Tc lymphocytes (Figure 4A, Supplementary Figure 5B).

The percentage of cells expressing CD71 and the cell surface expression of CD71 decreased upon Gal-14, but not Gal-13 treatment on both Th (% control: 96.9 ± 0.8%, Gal-14: 91 ± 1.3%, *p* < 0.05; RMFI control: 175.1 ± 17, Gal-14: 129 ± 6.1, *p* < 0.05) and Tc (% control: 97 ± 0.5%, Gal-14: 95 ± 1.1%, *p* < 0.05; RMFI control: 191.4 ± 26, Gal-14: 129.3 ± 33.2, *p* < 0.01) lymphocytes (Figure 4B, Supplementary Figure 5B).

Neither the percentage of CD25 nor of HLA-DR positive cells changed upon galectin treatment (Figures 4C,D, Supplementary Figure 5B). However, cell surface expression of CD25 increased upon Gal-13 treatment (Th RMFI control: 59.1 ± 8.1, Gal-13: 161 ± 30, *p* < 0.05; Tc RMFI control: 42.7 ± 9.6, Gal-13: 103.5 ± 24, *p* < 0.05), and tended to increase upon Gal-14 (RMFI Th: 110.6 ± 23.7; Tc: 64.4 ± 9.1) treatment in both T cell populations (Figure 4C). Of note, treatment of non-activated cells with Gal-13 or Gal-14 did not change the expression of these activation markers (Supplementary Figure 5A).

### Gal-13 and Gal-14 Induce Il-8 Secretion of T Cells

**Figure 5:**
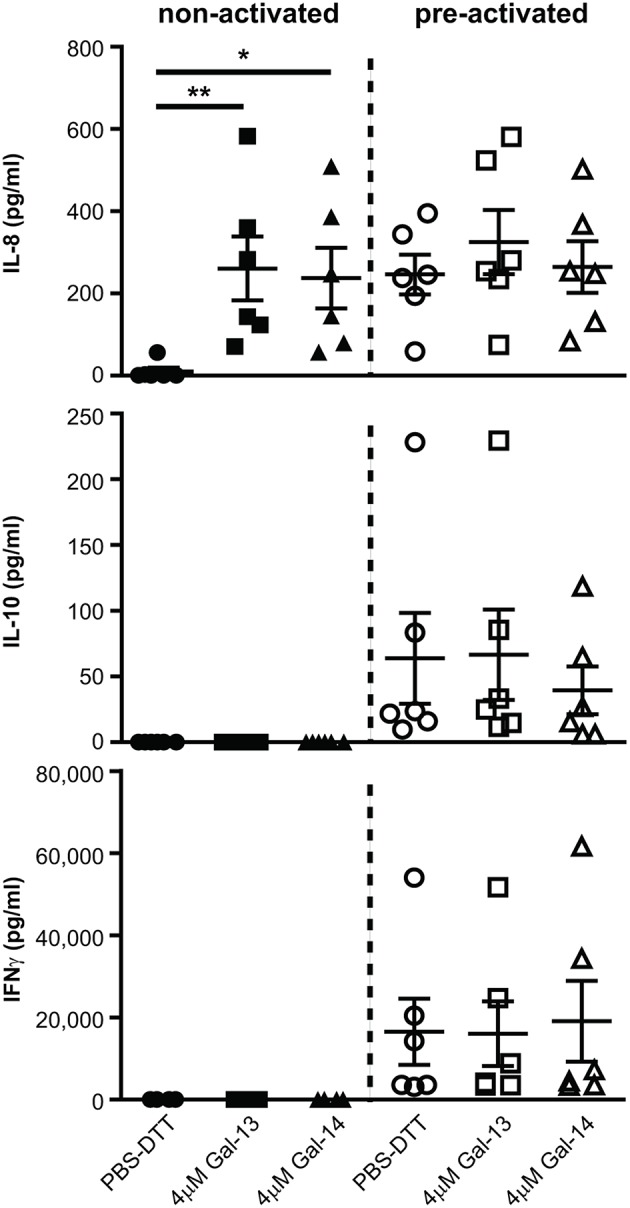
Galectin-13 and galectin-14 induce IL-8 production in resting T lymphocytes. T lymphocytes after negative selection were kept in culture for 72 h in the presence (pre-activated) or absence (non-activated) of anti-CD3/CD28 microbeads, then treated with 4 μM Gal-13 or Gal-14 for 24 h. Supernatants were collected and IL-8, IL-10, and IFNγ cytokines were measured by flow cytometry using LEGENDplex assays. Graphs show calculated cytokine concentrations as mean ± SEM. Repeated ANOVA with Dunnett's *post-hoc* test was used for the comparison of the non-treated group with Gal-13/Gal-14-treated groups (^*^p < 0.05, ^**^p < 0.01). Six non-pregnant female donors were included in each group.

Next, we sought to explore whether T lymphocytes contribute to the altered cytokine production, previously measured in PBMCs (114). IL-1β and IL-6 concentrations were below the detection limit in cell culture supernatants (data not shown). Surprisingly, IL-8 production was increased upon treatment with either Gal-13 (260.7 ± 78 pg/mL, *p* < 0.01) or Gal-14 (237.4 ± 73.5 pg/mL, *p* < 0.05) compared to the control (10.6 ± 9.2 pg/mL), when T cells were not activated. In the case of activation through CD3 and CD28, galectins could not further increase IL-8 production (Figure 5). Neither IL-10 nor IFNγ production changed upon galectin treatment (Figure 5).

## Discussion

### Principal Findings of the Study

(1) Gal-13 and Gal-14 are mainly expressed by the syncytiotrophoblast at the maternal-fetal interface in the first trimester, stronger than in the third trimester of pregnancy; (2) the syncytiotrophoblastic expression of both Gal-13 and Gal-14 is down-regulated in miscarriages compared to first trimester control placentas; (3) recombinant Gal-13 and Gal-14 differentially bind to peripheral blood T cell populations, predominantly to Tc over Th cells; (4) Gal-13 and Gal-14 induce the apoptosis of both T cell populations regardless of their activation status; (5) Gal-14 decreases the cell surface expression of CD71, Gal-13 and Gal-14 increase the cell surface expression of CD95 and Gal-13 increases the cell surface expression of CD25 on T cells; and (6) non-activated T cells produce larger amounts of IL-8 in the presence of Gal-13 or Gal-14.

### Placental Galectin-13 and Galectin-14 Expression Is Decreased in Miscarriage

This is the first study to characterize the simultaneous expression of Gal-13 and Gal-14 in first-trimester placentas in healthy and complicated pregnancies. We found that these placenta-specific galectins are mainly expressed by the syncytiotrophoblast at the lining of the maternal-fetal interface, similar to these galectins that are expressed in the placenta in the third trimester (53, 106, 110, 113). The expression of these galectins in the syncytiotrophoblast is developmentally regulated during trophoblast differentiation by transcription factors binding to non-coding elements upfront of these galectin genes on Chromosome 19, a process that emerged in anthropoid primates (110). This is particularly interesting from an immunological point of view given that these anthropoids had a long gestation, which necessitated additional immune tolerance mechanisms at their maternal-fetal interface to prevent fetal rejection (53). In this latter evolutionary and immune functional study, we proposed that the emergence of these galectins in anthropoid primates provided additional immune tolerance toward the fetus. Previous studies on placentas delivered by women with preeclampsia, a severe obstetrical syndrome with an immune rejection component, revealed the down-regulation of Gal-13 and Gal-14 placental expression, suggesting that this phenomenon may be linked to altered immune tolerance (81, 110, 113, 129).

Herein, we report for the first time that the placental expression of Gal-13 and Gal-14 is also decreased in first-trimester miscarriages. As most of these cases have a normal karyotype, our finding suggests that altered maternal-fetal immune tolerance is not closely associated with chromosomal abnormalities but can be a separate underlying mechanism for miscarriages. Indeed, recent publications presented that miscarriages are associated with immune etiologies (150), where the development of the fetus and placenta is affected by either auto- or alloimmune rejection-type activity (151). Of interest, the expression of other galectins, although in modest extent, is also decreased at the maternal-fetal interface in spontaneous and recurrent miscarriages, including Gal-1, Gal-2, Gal-7, Gal-9, and Gal-10 (152–154). Furthermore, in good accordance, serum concentrations of Gal-1 and Gal-9 were also found to be decreased in miscarriage (99, 154, 155). An elegant study revealed that Gal-1 has pivotal functions supporting maternal-fetal immune tolerance and its decreased expression leads to fetal loss in a mouse model. Gal-1 prevents fetal loss and restores tolerance through multiple mechanisms, including the induction of tolerogenic dendritic cells, which, in turn, promotes the expansion of IL-10-secreting regulatory T cells *in vivo* (107). On the other hand, Gal-9 was found to exert its functions in non-pregnant and pregnant states on NK cells, T cells, and B cells (101, 102, 147, 156–159). In addition, Gal-9 promotes trophoblast invasion in a Tim-3 dependent manner (154). Consistently, a higher proportion of decidual T cells that express activation markers (CD25, and CD69) was found in spontaneous abortion than in elective termination of pregnancy, and decidual lymphocytes from spontaneous abortion increased the apoptosis of trophoblast cells (160). Since Gal-13 and Gal-14 are only expressed in anthropoid primates, it is not possible to investigate the role of these proteins in knock-out mammalian models *in vivo*. Nevertheless, we can conclude that several galectins, including Gal-13 and Gal-14, potentially act in concert and play a role in maintaining pregnancy and that their lower expression at the maternal-fetal interface in early pregnancy may lead to an immune imbalance that interferes with implantation, trophoblast invasion, and placentation, leading to fetal rejection and miscarriages.

### Galectin-13 and Galectin-14 Promote Apoptosis of T Cells

The majority of the galectin family regulates adaptive immune responses through the induction of T cell apoptosis, which then leads to a shift in the innate/adaptive, Th1/Th2, and Th17/Treg immune balances (161–163). Similarly, we reported that exogenously added Gal-13 and Gal-14 are able to induce the apoptosis of activated T cells to a similar extent as Gal-1 (53). In accord with these findings, the interesting study from Kliman et al. (114) showed that Gal-13 is secreted from the syncytiotrophoblast and forms perivenous aggregates in the decidual extracellular matrix in the first trimester. These Gal-13 aggregates, found around decidual veins, were associated with T cell-, neutrophil-, and macrophage-containing “decidual zones of necrosis” (ZONEs), in which apoptotic T cells were also found. Based on these findings in normal pregnancies, and also based on cases in which fewer ZONEs and apoptotic T cells were found in association with very low serum Gal-13 levels, the authors hypothesized that Gal-13 is a key placental protein that downregulates maternal immune responses in the first-trimester decidua to avoid rejection of invasive trophoblasts at the maternal-fetal interface, and that low Gal-13 expression leads to heightened immune responses and impaired trophoblast invasion. It is possible that in cases where this mechanism is very defective (e.g., due to the concerted downregulation of Gal-13, Gal-14, Gal-1, and other immunoregulatory molecules in the placenta or decidua), pregnancies will be miscarried.

To better elucidate the role of Gal-13/Gal-14 in the regulation of T cells, we further characterized the pro-apoptotic effect of these galectins on T lymphocytes. We found that both Gal-13 and Gal-14 increased the rate of late-apoptotic T lymphocytes with ~5–10%, which was not affected by the activation status of the cells, while other galectins promote the apoptosis of only activated leukocytes (164, 165). Our result suggests that Gal-13 and Gal-14 have a basic pro-apoptotic activity on T cells. Of note, Tc cells bound more galectins than Th cells and Tc cells were more susceptible to Gal-13/Gal-14 induced apoptosis than Th cells, which may be related to the differential glycosylation pattern on these two T cell populations. This phenomenon has not been deeply explored in other galectins, which, however, were studied for their effects on different Th subsets. For example, Gal-1 selectively induced the apoptosis of pro-inflammatory Th1 and Th17 cell subsets, but not of naïve, Th2, or Treg cells (166). Moreover, Gal-9 induced the apoptosis of Th1 cells (147) in a Tim-3 dependent manner. Our results warrant further characterization of the pro-apoptotic effects of Gal-13 and Gal-14 on different Th subsets and determination of glycophenotype on T cell populations.

Apoptotic cell death in activated T cells is mediated by signaling through the activation marker CD95 (Fas), following binding to its ligand CD95L/FasL (167). In addition, another T cell activation marker CD25 (Il-2Rα), important for T cell proliferation, is also involved in this process by increasing the expression of CD95 (168). Herein, we found that the cell surface expression of CD95 and CD25 are increased upon Gal-14 and/or Gal-13 treatment. This is important since activated T cells are more prone to apoptosis (169); thus, Gal-13 and Gal-14 may increase the sensitivity of activated T cells to die by the activation-induced cell death. This is concordant with an earlier study in which Gal-1 increased the percentage of Th1 cells expressing CD95, although the Gal-1-mediated apoptosis of these cells was independent of CD95 (149). Interestingly, we found decreased expression of another activation marker (CD71) on T cells treated with Gal-14. This is seemingly contradictory, however, CD71 transiently associates with the TCR in response to TCR engagement (170) and is an essential factor for proliferating T cells (171, 172). Thus, galectins may inhibit T cell proliferation, which still needs to be tested in later studies.

### Galectin-13 and Galectin-14 Regulate Cytokine Production of T Cells

Several galectins have been shown to alter cytokine production of immune cells. For example, Gal-1 induces IL-10 production in Treg cells (107, 173) and Gal-9 promotes IL-2 and IFNγ production in T cells (164). Herein, we found in the applied experimental settings that Gal-13 and Gal-14 did not alter most pro-inflammatory (IFNγ, IL-1β, and IL-6) or anti-inflammatory (IL-10) cytokine production of T cells; however, both of these galectins induced IL-8 production in non-activated T cells. This is particularly interesting since IL-8 exerts a pro-angiogenic effect on endothelial cells by decreasing the apoptosis of endothelial cells and increasing their proliferation and capillary formation (174). In addition, a novel neutrophil population was identified by recent studies in second-trimester human deciduas, which promoted *in vitro* angiogenesis in an IL-8-dependent manner (175, 176). Furthermore, decidual NK cell subsets release significant amounts of pro-angiogenic factors, such as VEGF and IL-8, necessary for spiral artery formation during decidualization (177–179). Of note, the pro-angiogenic effects of other galectins during gestation have been discovered, as reviewed recently (180). Therefore, it is tempting to speculate that Gal-13 and Gal-14 may induce angiogenesis at the maternal-fetal interface through increasing IL-8 production of T cells. This finding is related to the *in vivo* vasodilator effect of Gal-13 (181–184). In this context, reduced Gal-13 and Gal-14 expression may play a role in the disturbed vascular changes in preterm preeclampsia (58, 185–194). All our data discussed above support the idea that Gal-13 and Gal-14 also have immunoregulatory and vascular effects, as found for galectin-1 or galectin-3 (195, 196). Since the immunomodulatory effects of Gal-13 and Gal-14 could be observed on a broad scale, changing the habit of adaptive immune cells may affect innate immune cells, as well. More experiments are warranted in this direction to comprehensively elucidate the effects of these galectins at the maternal-fetal interface.

### Strengths and Limitations of the Study

The strengths of the study are as follows: (1) strict clinical definitions and homogenous patient groups; (2) standardized, quick placental sample collection during pregnancy terminations; (3) standardized histopathological examination of the placentas based on international criteria; (4) protein expression profiling on placentas with tissue microarray and immunostaining followed by semiquantitative immunoscorings and statistical analysis; (5) expression and purification of large amounts of recombinant galectins with standardized methods; and (6) an array of functional experiments with primary cells and recombinant proteins.

Limitations of the study are as follows: (1) the relatively modest number of cases in each patient group due to the strict clinical and histopathological inclusion criteria used for patient enrollment. On the other hand, this was one of the most important strengths of our study; (2) for *in vitro* experiments, only non-pregnant donors were included in the study given the conditions in our patient recruitment. However, this was also a value of our study, since we used a “naïve” population of immune cells to test the effects of Gal-13 and Gal-14, while experiments with PBMCs isolated from the peripheral circulation or decidua of pregnant women pre-exposed to placental Gal-13/Gal-14 might not have revealed the true effects of these molecules. Nevertheless, our results warrant further characterization of the effects of Gal-13 and Gal-14 on peripheral blood and decidual leukocytes isolated from pregnant women, as pregnancy hormones, especially estrogen and progesterone, may impact glycosylation pattern and galectin-biding capacity of these cells; (3) Gal-13 and Gal-14 concentrations applied in our *in vitro* experiments were supraphysiologic, similar to experimental settings in previous studies on the functional effects of galectins (7, 165). The use of higher galectin concentrations is due to the fact that blood concentrations of galectins do not reflect their effective local/cell surface concentrations. In fact, these studies usually applied recombinant galectins between 10 and 100 μg/mL, representing a reasonable range of the local galectin concentration expected in the tissues (197). Another technical reason to use higher galectin concentrations is to prevent their subunit dissociation in the solvent that contains DTT (198); and (4) the evaluation of blood concentrations of Gal-13/Gal-14, which may change in parallel with their placental dysregulation in miscarriages, as also seen in the case of Gal-1 (99, 153, 155, 184), was beyond the scope of this study, but our results warrant further investigation.

## Concluding Remarks

The causes and consequences of the down-regulation of placental Gal-13 and Gal-14 expression in miscarriages still have to be uncovered by later functional studies. This work suggests that Gal-13 and Gal-14 down-regulate adaptive immune responses at the maternal-fetal interface through T cell apoptosis, and that their impaired expression leads to fetal rejection in miscarriages. Another process in which these galectins may function is angiogenesis, which is altered in both miscarriage and preeclampsia, in which Gal-13 and Gal-14 expression is decreased. Since galectins have pleiotropic functions on various immune and non-immune cells given their promiscuous binding to various cell surface receptors via glycan binding, we envision that both actions may be functional in human pregnancy. In conclusion, our results suggest that Gal-13 and Gal-14 provide an immunoprivileged environment at the maternal-fetal interface, already in early pregnancy, either through down-regulating maternal immune responses or via the support of placental development, and their reduced expression is related to the immune pathology of miscarriages.

## Data Availability

All datasets generated for this study are included in the manuscript and/or the Supplementary Files.

## Ethics Statement

Clinical sample and data collection were approved by the Health Science Board of Hungary (ETT-TUKEB 4834-0/2011-1018EKU). Written informed consent was obtained from women prior to sample collection and the experiments conformed to the principles set out in the World Medical Association Declaration of Helsinki. Specimens and data were stored anonymously.

## Author Contributions

AB, IH, SH, JM, SR, EP, and NT conceptualized study and designed research. AB, DC, KJ, AK, EP, KP, NS, and ET performed research. SH, PH, HM, EP, ZP, RR, NT, and PZ contributed new reagents, analytic tools, and clinical specimens. AB, DC, OE, JM, EP, SR, AT, and NT analyzed and interpreted data. All authors contributed to the writing of the paper.

### Conflict of Interest Statement

HM is the CEO and Chairman of TeleMarpe Ltd. and is a consultant of Hy Laboratories. The remaining authors declare that the research was conducted in the absence of any commercial or financial relationships that could be construed as a potential conflict of interest.
