# Supplementary material for: Placental Galectins Are Key Players in Regulating the Maternal Adaptive Immune Response
Source: Front Immunol. 2019 Jun 19;10:1240. doi: 10.3389/fimmu.2019.01240 (PMC6593412; doi:10.3389/fimmu.2019.01240)
Supplement: Supplementary file 1 [file Presentation_1.pdf]

Supplementary Material

Supplementary Table 1. Immunohistochemistry conditions

**Primary antibody (concentration, distributor)**	**Secondary antibody (concentration, distributor)**	**Detection antibody (distributor)**	**Detection system (distributor)**
Mouse monoclonal anti-human galectin-13 antibody (2μg/ml) (Hy Laboratories)	-	Rabbit anti-mouse IgG and anti-rabbit poly-HRP-IgG (Novocastra Laboratories)	Novolink Polymer Detection System (Novocastra Laboratories)
Recombinant anti-human galectin-14 antibody with His_6-tag (0.65μg/ml) (Bio-Rad)	Monoclonal anti-His-tag antibody (5μg/ml) (Merck-Millipore)	Rabbit anti-mouse IgG and anti-rabbit poly-HRP-IgG (Novocastra Laboratories)	Novolink Polymer Detection System (Novocastra Laboratories)

Supplementary Table 2. Western blot conditions

**Primary antibody (concentration)**	**Primary antibody source**	**HRP-antibody (dilution)**	**HRP-antibody source**
Mouse monoclonal anti-human galectin-13 antibody (1μg/ml)	Hy Laboratories	Goat anti-mouse IgG (1:5,000)	ThermoFisher Scientific
Recombinant anti-human galectin-14 antibody (1μg/ml)	Bio-Rad	Goat anti-human IgG $F(ab')_2$ (1:10,000)	Bio-Rad

Supplementary Table 3. Flow cytometry antibodies/reagents

Antibody/Reagent	Clonality	Amount/test	Source (Cat. #)
**human FcR blocking reagent**	-	2µl	Miltenyi Biotec (130-059-901)
**Gal-13-CF488 recombinant protein**	-	5µg	MBP-tag modified version of the Gal-13 expression plasmid which was provided by Prof. R. Romero (PRB, NIH)
**Gal-14-CF488 recombinant protein**	-	5µg	MBP-tag modified version of the Gal-13 expression plasmid which was provided by Prof. R. Romero (PRB, NIH)
**anti-human CD3-APC**	monoclonal	2.5µl	Biolegend (317318)
**anti-human CD4-PerCP/Cy5.5**	monoclonal	2.5µl	Biolegend (317428)
**anti-human CD8-FITC**	monoclonal	2.5µl	Biolegend (301006)
**anti-human CD8-APC/Fire750**	monoclonal	2.5µl	Biolegend (301066)
**mouse IgG1-PE**	monoclonal	2.5µl	Biolegend (400114)
**mouse IgG2a-PerCP/Cy5.5**	monoclonal	2.5µl	Biolegend (400252)
**anti-human CD25-PE**	monoclonal	2.5µl	Biolegend (356104)
**anti-human CD71-PerCP/Cy5.5**	monoclonal	2.5µl	Biolegend (334114)
**anti-human CD95-PE**	monoclonal	5µl	BD Biosciences (555674)
**anti-human HLA-DR-PerCP/Cy5.5**	monoclonal	2.5µl	Biolegend (307630)
**Annexin V-PE**	-	5µl	ThermoFisher Scientific (88-8102-74)
**7-AAD**	-	5µl	ThermoFisher Scientific (88-8102-74)

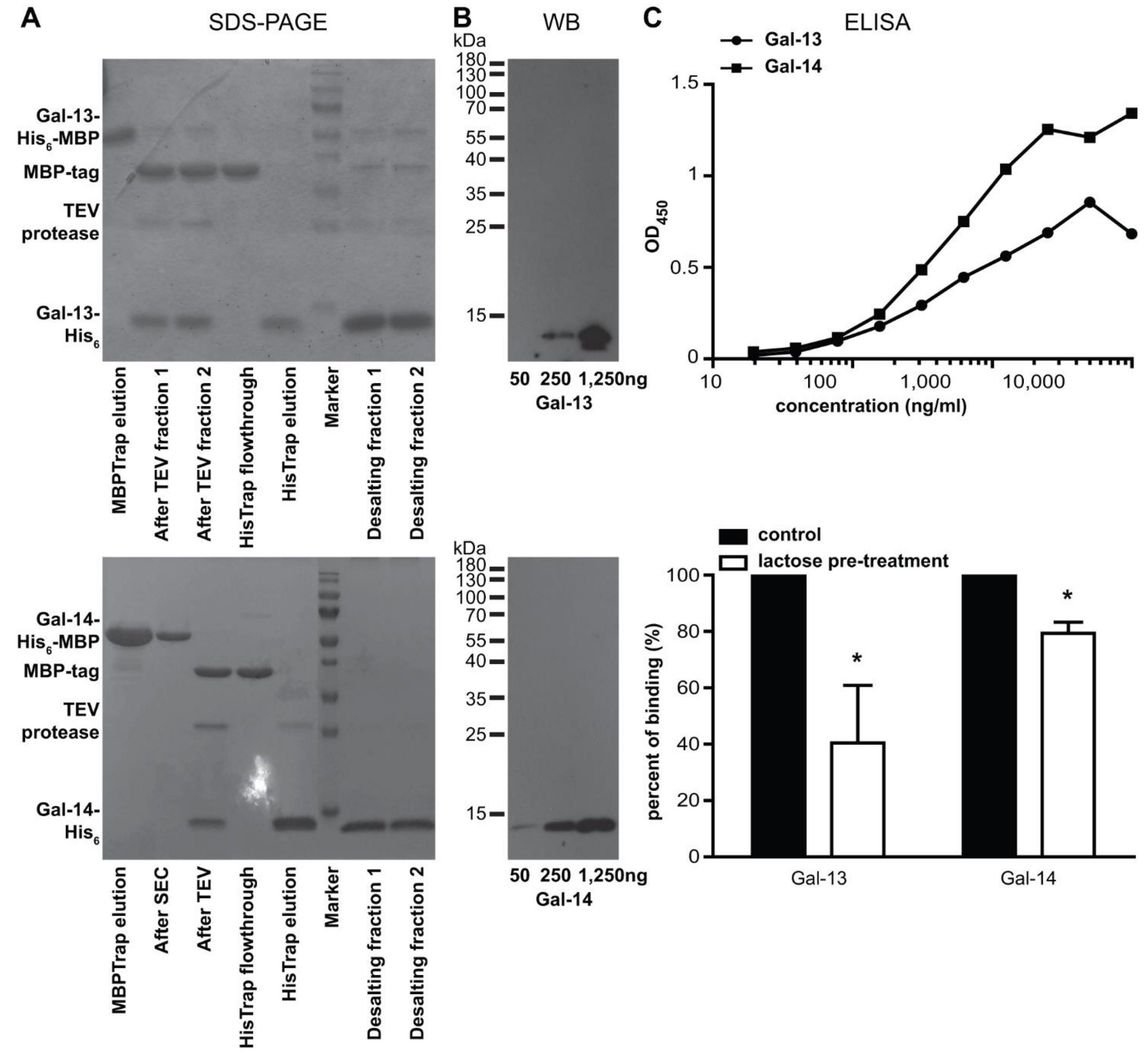

Supplementary Figure 1. Production of recombinant galectins. (**A**) Recombinant Gal-13 and Gal-14 were expressed in ClearColi BL21. Purification steps were monitored by 15% SDS-PAGE: 1) affinity chromatography on MBPTrap HP column; 2) size exclusion chromatography (SEC) for Gal-14; 3) MBP cleavage by TEV protease; 4) affinity chromatography on HisTrap HP column; 5) desalting and buffer exchange. Recombinant galectins with His_6- and MBP-tags have approx. 59 kDa. The MBP-tag is ~42 kDa, TEV protease is ~26 kDa, and the TEV-cleaved galectins with the His_6-tag are ~17 kDa in the monomeric form. Representative gels for Gal-13 (upper image) or Gal-14 (lower image) are displayed. (**B**) Purified galectins were subjected to Western blot analysis with specific antibodies to Gal-13 or Gal-14 and subsequently with HRP-conjugated secondary antibodies. Representative blots for Gal-13 (upper image) or Gal-14 (lower image) are shown, with specific bands at ~17 kDa. (**C**) Binding of purified galectins to asialofetuin (ASF) was assayed by ELISA. ASF was absorbed to microtiter plates. After blocking, serially diluted Gal-13 or Gal-14 was added, followed by incubation with anti-His_6-HRP. TMB served as the enzyme substrate (450 nm, reference filter: 620 nm). The upper graph is representative of three independent measurements. Binding of 50 μg/ml Gal-13 or Gal-14 to ASF with or without (control, taken as 100%) pre-treatment on lactose-agarose beads is displayed on the lower graph as mean ± SEM of three independent measurements. One sample t-test was used for the comparison of the binding of Gal-13 and Gal-14 to ASF with or without lactose pre-treatment (*$p < 0.05$). Horseradish peroxidase, HRP; maltose-binding protein, MBP; Tobacco Etch virus, TEV.

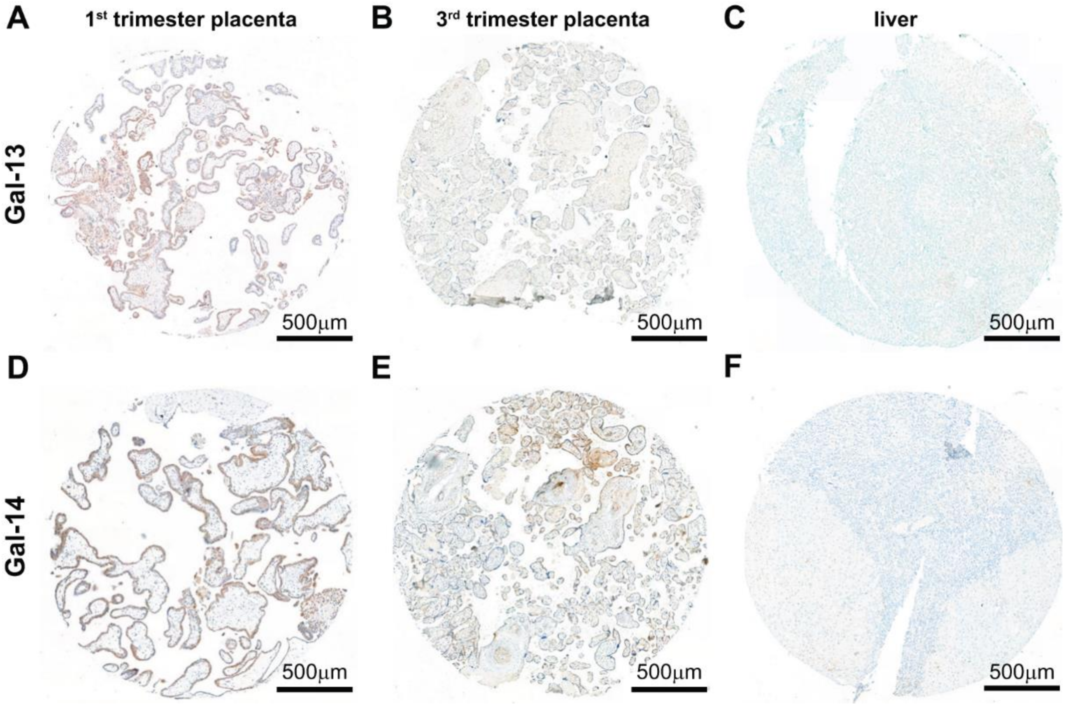

Supplementary Figure 2. Galectin-13 and galectin-14 expression in first trimester placentas. 2 mm diameter representative TMA cores are presented from first trimester placentas **(A, D)**, positive control third trimester placentas **(B, E)** or negative control livers (**C, F**). TMAs were either stained for Gal-13 (**A-C**) or Gal-14 (**D-F**) using specific monoclonal antibodies. Chorionic villi exhibited more intense syncytiotrophoblast cytoplasmic staining in the first trimester than in the third trimester for both Gal-13 and Gal-14, while livers did not stain. Hematoxylin counterstain, 40x magnifications. Tissue microarray, TMA.

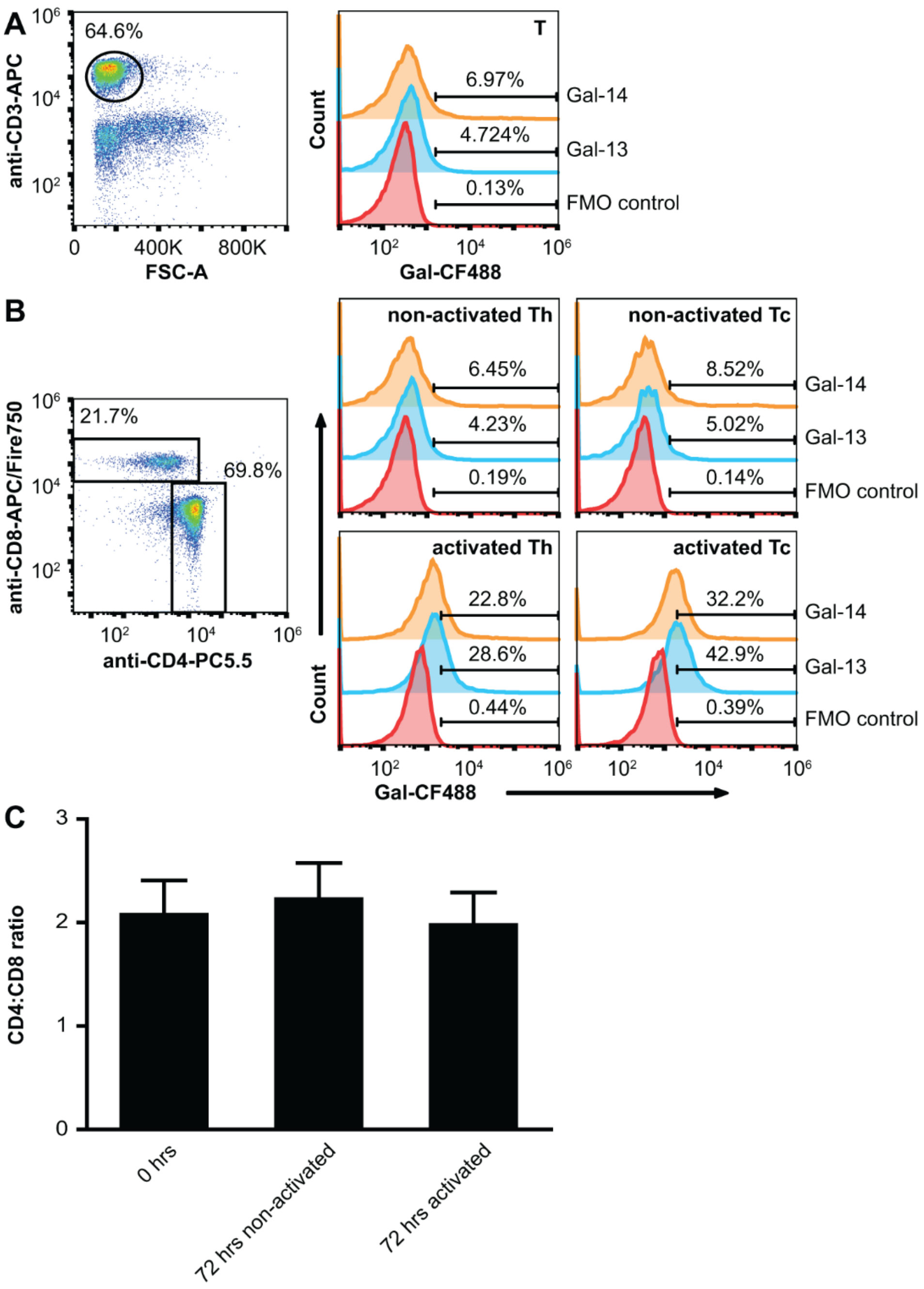

Supplementary Figure 3. Galectin-13 and galectin-14 bind to T lymphocytes. (**A**) To detect binding of Gal-13 or Gal-14 to human T lymphocytes, freshly isolated PBMCs (**A**) or PBMCs, kept in culture for 72 h in the presence (activated) or absence (non-activated) of anti-CD3/CD28 microbeads (**B**), were incubated with 4 µM Gal-13-CF488 or Gal-14-CF488 for 45 min on ice. Cells were also stained for CD3 (anti-CD3-APC), CD4 (anti-CD4-PC5.5), and CD8 (anti-CD8-APC/Fire750), in order to distinguish between helper (Th) and cytotoxic (Tc) T lymphocytes during flow cytometric measurements. (**A**) A dot plot showing gating on all T cell, and a representative histogram of Gal-13 (blue) or Gal-14 (orange) binding to T lymphocytes are displayed (red: FMO control). (**B**) A dot plot showing the gating on Th and Tc cell, and representative histograms of Gal-13 (blue) or Gal-14 (orange) binding to Th or Tc lymphocytes upon activation is displayed (red: FMO control). (**C**) The graph shows CD4:CD8 ratio of freshly isolated PBMCs or PBMCs activated or not with anti-CD3/CD28 microbeads for 72 h (mean ± SEM). Repeated ANOVA with Tukey's post hoc test was used for the comparison of groups. Fluorescence minus one, FMO; peripheral blood mononuclear cells, PBMCs.

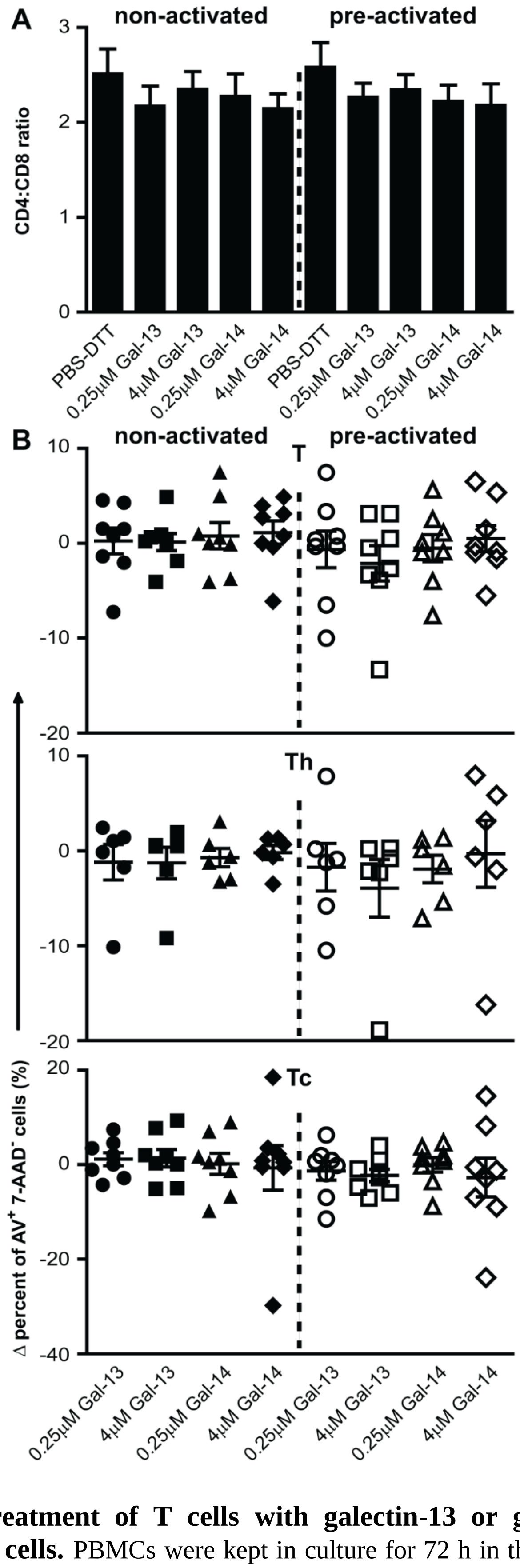

Supplementary Figure 4. Treatment of T cells with galectin-13 or galectin-14 did not alter the percentage of early apoptotic cells. PBMCs were kept in culture for 72 h in the presence (activated) or absence (non-activated) of anti-CD3/CD28 microbeads, then treated with Gal-13 or Gal-14 for 24 h. Cells were stained with Annexin V-PE and 7-AAD and were also stained for CD3 (anti-CD3-APC), and CD8 (anti-CD8-FITC), in order to distinguish between helper (Th) and cytotoxic (Tc) T lymphocytes during flow cytometric measurements. (**A**) CD4:CD8 ratio upon different treatments (mean ± SEM) is displayed, using repeated ANOVA with Tukey's post hoc for the comparison of groups. (**B**) Graphs show the Δ percentage of single positive (Annexin V-PE^{+} 7-AAD^{-}) cells as mean ± SEM, using one sample t-test for the comparison of galectin-treated groups with the PBS-DTT-treated group of non-activated or pre-activated cells. Six-eight non-pregnant female donors were included in each group. Peripheral blood mononuclear cells, PBMCs.

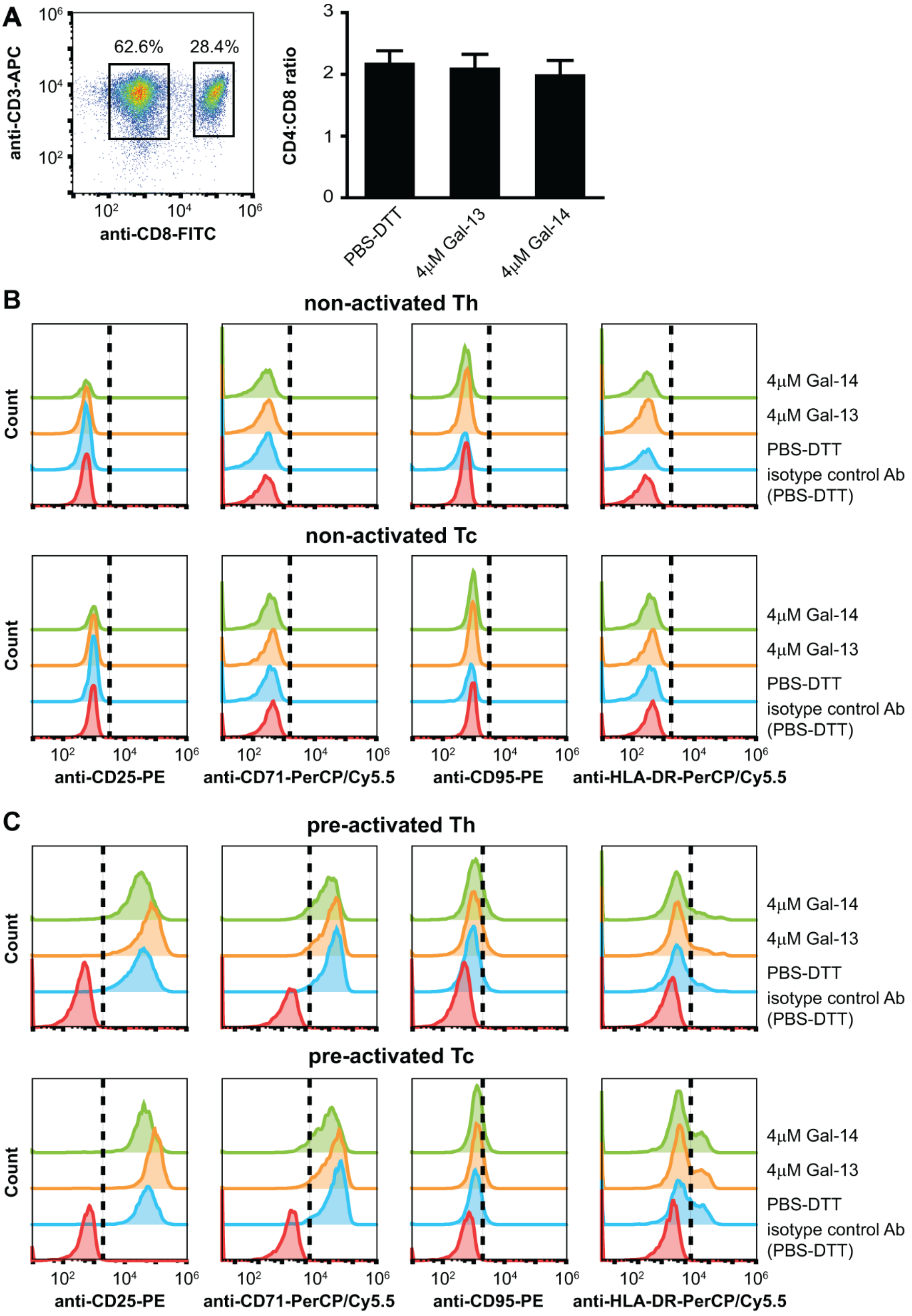

Supplementary Figure 5. Activation marker expression on T lymphocytes upon galectin treatment. PBMCs were kept in culture for 72 h in the presence or absence of anti-CD3/CD28 microbeads, then treated with Gal-13 or Gal-14 for 24 h. To detect cell surface expression of CD25, CD71, CD95, HLA-DR activation markers on T lymphocytes, cells were stained with anti-CD25-PE and anti-CD71-PerCP/5.5, or anti-CD95-PE and anti-HLA-DR-PerCP/5.5. Cells were also stained for CD3 (anti-CD3-APC), and CD8 (anti-CD8-FITC) in order to distinguish between helper (Th) and cytotoxic (Tc) T cells during flow cytometric measurements. Representative dot plot of CD3 and CD8 staining, CD4:CD8 ratio of pre-activated and Gal-treated cells (mean ± SEM, **A**), and demonstrative histograms of the results of 2 (non-activated, **B**) or 4-6 (pre-activated, **C**) non-pregnant female donors are displayed. Dashed lines show the border between negative and positive populations. Repeated ANOVA with Tukey's post hoc test was used for the comparison of CD4:CD8 ratio upon different treatments. Human leukocyte antigen DR isotype, HLA-DR; peripheral blood mononuclear cells, PBMCs.
